# Therapeutic Potential of Exogenous Ketone Supplement Induced Ketosis in the Treatment of Psychiatric Disorders: Review of Current Literature

**DOI:** 10.3389/fpsyt.2019.00363

**Published:** 2019-05-23

**Authors:** Zsolt Kovács, Dominic P. D’Agostino, David Diamond, Mark S. Kindy, Christopher Rogers, Csilla Ari

**Affiliations:** ^1^Savaria Department of Biology, ELTE Eötvös Loránd University, Savaria University Centre, Szombathely, Hungary; ^2^Department of Molecular Pharmacology and Physiology, Laboratory of Metabolic Medicine, Morsani College of Medicine, University of South Florida, Tampa, FL, United States; ^3^Institute for Human and Machine Cognition, Ocala, FL, United States; ^4^Department of Psychology, Hyperbaric Neuroscience Research Laboratory, University of South Florida, Tampa, FL, United States; ^5^Department of Pharmaceutical Sciences, College of Pharmacy, University of South Florida, Tampa, FL, United States; ^6^James A. Haley VA Medical Center, Tampa, FL, United States; ^7^Shriners Hospital for Children, Tampa, FL, United States

**Keywords:** psychiatric diseases, exogenous ketone supplements, ketosis, mitochondrial dysfunction, inflammation

## Abstract

Globally, psychiatric disorders, such as anxiety disorder, bipolar disorder, schizophrenia, depression, autism spectrum disorder, and attention-deficit/hyperactivity disorder (ADHD) are becoming more prevalent. Although the exact pathological alterations are not yet clear, recent studies have demonstrated that widespread changes of very complex metabolic pathways may partially underlie the pathophysiology of many psychiatric diseases. Thus, more attention should be directed to metabolic-based therapeutic interventions in the treatment of psychiatric disorders. Emerging evidence from numerous studies suggests that administration of exogenous ketone supplements, such as ketone salts or ketone esters, generates rapid and sustained nutritional ketosis and metabolic changes, which may evoke potential therapeutic effects in cases of central nervous system (CNS) disorders, including psychiatric diseases. Therefore, the aim of this review is to summarize the current information on ketone supplementation as a potential therapeutic tool for psychiatric disorders. Ketone supplementation elevates blood levels of the ketone bodies: D-β-hydroxybutyrate (βHB), acetoacetate (AcAc), and acetone. These compounds, either directly or indirectly, beneficially affect the mitochondria, glycolysis, neurotransmitter levels, activity of free fatty acid receptor 3 (FFAR3), hydroxycarboxylic acid receptor 2 (HCAR2), and histone deacetylase, as well as functioning of NOD-like receptor pyrin domain 3 (NLRP3) inflammasome and mitochondrial uncoupling protein (UCP) expression. The result of downstream cellular and molecular changes is a reduction in the pathophysiology associated with various psychiatric disorders. We conclude that supplement-induced nutritional ketosis leads to metabolic changes and improvements, for example, in mitochondrial function and inflammatory processes, and suggest that development of specific adjunctive ketogenic protocols for psychiatric diseases should be actively pursued.

## Introduction

With an increasing global prevalence, psychiatric disorders can present as serious medical conditions composed of emotional, cognitive, social, behavioral, and functional impairments (1). Lifetime onset of major depressive disorders in the general population is up to 11–16% (2, 3), with bipolar disorder present in 1% (4, 5), schizophrenia in 1% (6, 7), and anxiety disorder in 5–31% (1). In relation to attention-deficit/hyperactivity disorder (ADHD), worldwide prevalence of this disease in children/adolescence and adults is about 5.3% and 2.5%, respectively (8, 9), while about 1 in 68 children were diagnosed with autism in the United States in 2012 (10). It has been demonstrated that not only genetic factors but also environmental factors (e.g., infections, early traumas, and drugs), age, sociodemographic factors (e.g., ethnicity and socioeconomic status), and a complex interplay between these factors have a role in the pathophysiology of different psychiatric diseases, such as anxiety disorder (1, 11), bipolar disorder (5), schizophrenia (6, 12), major depressive disorder (2, 13, 14), autism spectrum disorder (15), and ADHD (16). Close association between different psychiatric disorders, such as anxiety disorder and major depressive disorder, has been demonstrated (5, 17–21).

However, while symptoms, characteristics, and classification of different psychiatric disorders are adequately described (1, 5, 7, 15, 16, 22), the pathophysiology of psychiatric diseases is not yet fully understood. Nevertheless, recent studies have demonstrated that the disturbance in the monoaminergic (23–26) and other neurotransmitter systems (e.g., glutamatergic, purinergic, and GABAergic) (27–34), in addition to widespread changes of very complex and connected metabolic pathways, may partially explain the general condition. For example, it has been suggested that mitochondrial dysfunction could play a major role (35). Mitochondrial dysfunction may decrease energy/ATP production, impair calcium homeostasis, increase levels of reactive oxygen species (ROS), and alter apoptotic pathways, inflammatory processes, neurotransmission, synaptic plasticity, and neuronal activity and connectivity (35, 36). Moreover, changes in hypothalamic–pituitary–adrenal (HPA) axis activity were also demonstrated in patients with psychiatric diseases, in which alterations may influence mitochondrial functions: a chronic increase in glucocorticoid levels may decrease mitochondrial energy production (35, 37). Membrane lipid dysregulation may affect the levels of pro-inflammatory cytokines, as well as the function of mitochondria, ion channels, and neurotransmitter systems implicated in the pathophysiology of psychiatric diseases (38, 39). In addition, changes in membrane fatty acid composition may alter the function of different cell-surface receptors, ion pumps, and special enzymes, such as 5’-nucleotidase, adenylate cyclase, and Na^+^/K^+^-ATPase (38, 40). Increased activity of the inflammatory system and redox pathways may enhance oxidative and nitrosative stress, mitochondrial dysfunction, neurodegeneration and neuronal death, production of pro-inflammatory cytokines, and activity of the HPA axis, whereas it may decrease neurogenesis and serotonin levels (35, 37). In addition, functional brain imaging studies demonstrated abnormalities in regional cerebral glucose metabolism in the prefrontal cortex in patients with mood disorders, providing evidence of persistent hypometabolism, particularly in the frontal gyrus, in depressed patients (41). Recent transcriptomic, proteomic, and metabolomics studies have also highlighted an abnormal cerebral glucose and energy metabolism as one of the potential pathophysiological mechanisms of schizophrenia, raising the possibility that a metabolically based intervention might have therapeutic value in the management of the disease (42).

Consequently, different metabolic changes and their downstream effects may generate complex, interlinked molecular and cellular processes, which may lead to different psychiatric diseases. It can be concluded that alterations in multiple interactive metabolic pathways and their effects on different physiological processes may largely underlie the pathophysiology in patients with psychiatric diseases. Indeed, if defective metabolism is the cause of such pathologies, then utilization of therapies designed to address deficiencies of metabolism (known as metabolic therapies) would be a rational approach for the treatment of these diseases.

In a process known as ketogenesis, the ketone bodies [D-β-hydroxybutyrate (βHB), acetoacetate (AcAc), and acetone] are catabolized under normal physiological conditions by the liver from fatty acids as a source of fuel (43–45). Higher levels of ketones are produced during starvation, fasting, and neonatal development (46, 47). Moreover, although most of βHB, which is used as an energy source in the brain, is synthesized by the liver, ketone body synthesis and release by astrocytes have also been demonstrated (48, 49). Ketone bodies can transport to the bloodstream from the liver, cross the blood–brain barrier (BBB), enter brain cells through monocarboxylic transporters, convert to acetyl CoA in the mitochondria, and enter the Krebs cycle (43–44, 45, 50). Through this process, ketosis (increased ketone body levels in the blood) provides energy by metabolism of ketone bodies to acetyl-CoA and synthesis of ATP for cells in the central nervous system (CNS) (43, 51, 52). It has been demonstrated in animal—and/or human studies—that ketogenic diets and supplements may have metabolism-based therapeutic potential in the treatment of several diseases, such as Alzheimer’s disease (53–57), Parkinson’s disease (54, 58–60), glucose transporter type 1-deficiency syndrome (61–63), amyotrophic lateral sclerosis (60, 64), cancer (44, 58, 65, 66), epilepsy (54, 67, 68), schizophrenia (42, 69–74), anxiety (55, 75–77), autism spectrum disorder (78–81), and depression (69, 77, 82).

Ketogenic diets are high-fat, adequate protein and very low carbohydrate diets that may have an alleviating role on psychiatric diseases (69, 73), likely through bioenergetics, ketone metabolism, and signaling, as well as their effects on, for example, neuronal activity, neurotransmitter balance, and inflammatory processes (43, 52, 83–91). Strict patient compliance to the KD is the primary factor in achieving therapeutic ketosis, and this is often difficult or impossible in the psychiatric population (69). Therefore, the administration of exogenous ketone supplements including medium chain triglycerides (MCTs), ketone salt (KS), ketone ester (KE), and their combination with MCT oil (e.g., KSMCT) presents a strategy to circumvent dietary restriction to rapidly induce and sustain nutritional ketosis (65, 75, 84, 92). Ketone bodies not only enhance cell energy metabolism through anaplerotic effects but also suppress oxidative stress, decrease inflammatory processes, and regulate functions of ion channels and neurotransmitter systems (45, 93, 94)—all processes implicated in the pathophysiology of psychiatric diseases (1, 5, 6, 15, 16, 22). Therefore, the rationale exists for the use of exogenous ketone supplementation, which induces a nutritional ketotic state similar to that derived from the ketogenic diet and may mimic the effects of ketogenic diet on several CNS diseases through ketone body-evoked metabolic and signaling alterations (54, 55, 67, 75, 95–99) and epigenetic effects (100).

In contrast to diabetic ketosis, which can induce pathological levels of blood βHB (ranging >25 mM) and potentially lead to life-threatening acidosis, nutritional ketosis elevates blood βHB from the normal range (0.1–0.2 mM) to a safe and—in many cases—therapeutic range (1–7 mM: therapeutic ketosis) (44, 54, 101). While rigorous adherence to ketogenic diets is typically difficult to follow and requires clear medical guidance and strong motivation, consumption of exogenous ketogenic agents effectively induces ketosis with little difficulty (65, 75, 84, 92, 102). Moreover, prolonged consumption of ketogenic diets may generate side effects, such as weight loss, alteration of mentation, growth retardation, nephrolithiasis, nausea, constipation, gastritis, hyperlipidemia, hypoglycemia, hyperuricemia, and ulcerative colitis (44, 69, 103, 104). Consequently, developing a safer alternative method using ketone body precursors and exogenous ketone supplements, such as KSs or KEs, to circumvent dietary restriction is appealing.

Recent research has demonstrated that it is possible to rapidly increase and maintain blood levels of ketone bodies in a dose-dependent manner in both animals and humans (54, 84, 99) for the treatment of several CNS diseases (55, 64, 67, 75). Thus, it is possible that exogenous ketone supplementation-induced ketosis may be an effective therapeutic tool against psychiatric diseases. Indeed, exogenous ketone supplements have a modulatory influence on behavior and anxiolytic effect in animal studies (55, 75, 83). Moreover, in contrast to ketogenic diets, exogenous ketone supplements are relatively well-tolerated and can be formulated and titrated to minimize or avoid side effects (56, 65, 75, 84, 99, 105, 106).

There is limited evidence to support the beneficial effects of exogenous ketone supplements in psychiatric diseases at the moment [e.g., Refs. (55, 75, 76)], but the use of exogenous ketone supplements may be a viable alternative or adjuvant to pharmacotherapy in the treatment of these disorders. Consequently, in the following major section, we provide a short overview of the metabolism of exogenous ketone supplements, which results in rapid and safe mild therapeutic ketosis and, as a consequence, may be an alternative method to ketogenic diets for the treatment of psychiatric disorders. In the next major sections, therapeutic potential of exogenous ketone supplements in the treatment of each psychiatric disease is summarized. This is followed by a brief conclusions section with perspective and future outlook.

## Metabolism of Exogenous Ketone Supplements: Generation of Therapeutic Ketosis

**Figure 1:**
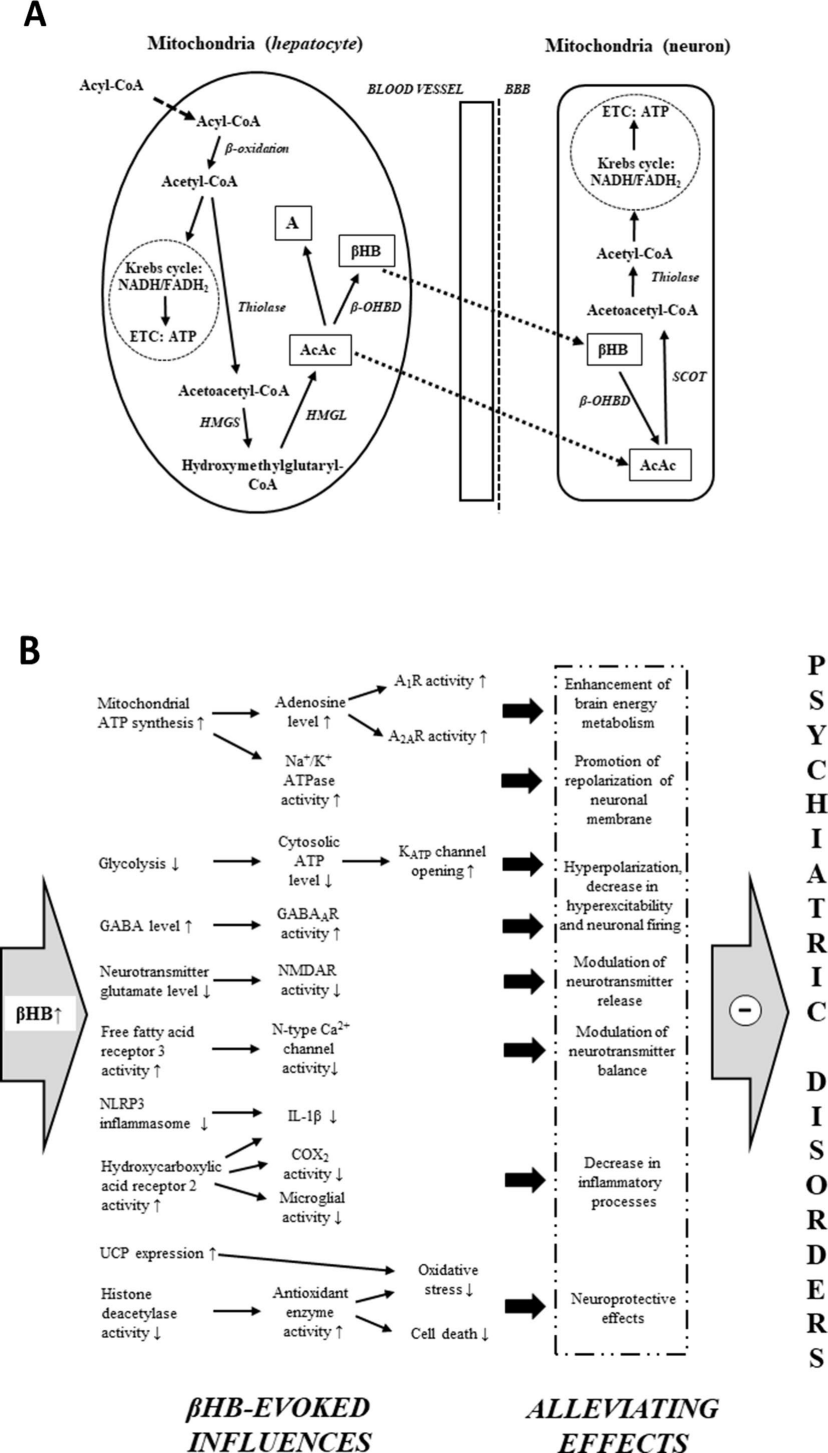
Mitochondrial ketone body metabolism: ketogenesis in liver cells (*hepatocytes*) and ketolysis in brain cells (neuron) **(A)**. Main βHB-evoked metabolic effects and their consequences, which may evoke alleviating effects on different psychiatric diseases **(B)** (see text for more detailed putative mechanisms by which βHB may evoke alleviating effects on psychiatric diseases). Abbreviations: A, acetone; A_1_R and A_2A_R, different types of adenosine receptors; AcAc, acetoacetate; ATP, adenosine triphosphate; BBB, blood–brain barrier; βHB, D-beta-hydroxybutyrate (R-3-hydroxybutyrate); β-OHBD, βHB dehydrogenase; COX-2, cyclooxygenase-2; ETC, electron transport chain; GABA, gamma-aminobutyric acid; HMGL, hydroxymethylglutaryl-CoA-lyase; HMGS, hydroxymethylglutaryl-CoA-synthase; IL-1β, interleukin-1β; NADH/FADH_2_, nicotinamide adenine dinucleotide/flavin adenine dinucleotide; NLRP3, NOD-like receptor pyrin domain 3; NMDAR, N-methyl-D-aspartate receptor; SCOT, succinyl-CoA:3-ketoacid CoA transferase; thiolase, acetoacetyl-CoA-thiolase; UCP, uncoupling proteins.

Under typical (high carbohydrate) diet conditions, glycogen-derived glucose is the main energy source of brain cells (43, 107). However, ketogenic diets, starvation, and fasting result in an increased reliance of the brain on fat-derived ketones for fuel (43, 44, 108). Free fatty acids are converted into acyl-CoA in the liver cells, and subsequently, acyl-CoA is metabolized to acetyl-CoA by mitochondrial β-oxidation (**Figure 1A**). Acetyl-CoA may generate energy (*via* Krebs cycle: tricarboxylic acid cycle/TCA cycle) or it gets converted into ketone bodies (43–44, 45, 50). As hepatocytes are not able to utilize the high levels of acetyl-CoA derived from ketogenic diet-, starvation-, and fasting-evoked increase in fatty acids, under these conditions, a large portion of acetyl-CoA can be converted to ketone bodies (44, 45, 107). Two acetyl-CoA molecules fuse into one acetoacetyl-CoA molecule by acetoacetyl-CoA-thiolase. Subsequently, hydroxymethylglutaryl-CoA-synthase (HMGS) condenses the third acetyl-CoA molecule with acetoacetyl-CoA to form hydroxymethylglutaryl-CoA (HMG-CoA) (this process, catalyzed by HMGS, is the rate-limiting step of ketogenesis) (43–44, 45, 50). AcAc is liberated from HMG-CoA by hydroxymethylglutaryl-CoA-lyase (HMGL). AcAc may reduce to βHB by a NADH molecule in a βHB dehydrogenase (β-OHBD) catalyzed reaction, or, in lesser amounts, a part of AcAc may metabolize to acetone by the spontaneous, non-enzymatic decarboxylation of AcAc (43–44, 45, 50). The major circulating water-soluble ketone body is βHB (44, 50). AcAc is a chemically unstable molecule, and acetone is a very volatile compound (eliminated mainly *via* respiration from the lungs) (44, 50). As the metabolic enzyme succinyl-CoA:3-ketoacid CoA transferase (SCOT) is not expressed in the liver, hepatocytes are not able to consume ketone bodies as an energy substrate (45, 50, 52); thus, AcAc and βHB can exit the liver, enter the bloodstream, and be distributed to various tissues, including the brain, after transport through monocarboxylate transporters (43–44, 45, 50). In the mitochondria of brain cells, ketone bodies are converted back to acetyl-CoA (**Figure 1A**) (43–44, 45, 50). As the first step of this metabolic pathway, βHB oxidizes to AcAc by NAD^+^ and β-OHBD. AcAc is then metabolized to acetoacetyl-CoA, which converts to two acetyl-CoA molecules (by SCOT and acetoacetyl-CoA-thiolase, respectively). Finally, acetyl-CoA molecules enter the Krebs cycle as an energy source for ATP synthesis (43–44, 45, 50).

While a ketogenic diet could potentially confer numerous benefits to patients suffering from psychiatric disorders, compliance to the diet would likely be low. Reasons include the lack of knowledge, support, palatability, and different adverse effects such as gastrointestinal side effects (69, 103, 104). Most importantly, ketogenic diets must continuously restrict carbohydrates (typically 20 g/day) to sustain ketogenesis through elevated long-chain fatty acid oxidation (109). Nevertheless, the production of ketone bodies from KSs or KEs (e.g., by liver alcohol dehydrogenase and/or hydrolysis in the small intestine) is not inhibited by carbohydrates; thus, ketone supplements may be usable while maintaining a normal diet (105) to generate therapeutic ketosis.

After consumption or gavage administration, KEs are fully hydrolyzed in the small intestine by esterases, which can be transported to the systemic bloodstream, and converted to 1,3-butanediol. Following this, 1,3-butanediol is metabolized to AcAc and βHB in the liver by alcohol and aldehyde dehydrogenase (106, 110, 111). Moreover, MCTs/MCT oils are hydrolyzed to medium chain fatty acids (e.g., decanoic and octanoic acid) by lipases in the gastrointestinal tract, which are metabolized to ketone bodies in the liver (112). Thus, similar to ketogenic diets, metabolism of exogenous ketone supplements may result in increased levels of blood ketone bodies, which may serve the energy needs of brain cells (**Figure 1A**). For example, KS supplementation significantly increased the mitochondrial activity of both β-OHBD and acetoacetyl-CoA-thiolase in the brain of rats (83), and oral administration of exogenous ketone supplements is able to evoke and maintain rapid and safe mild ketosis in both animals and human (54, 64, 65, 75, 84, 92, 99, 101, 106, 108).

Unfortunately, MCTs are often not well tolerated because of their gastrointestinal side effects (e.g., diarrhea, dyspepsia, and flatulence) and supplementation of MCTs generates relatively low levels of ketone bodies in the blood (113). Oral administration of KEs fully metabolizes to βHB and AcAc and, as a consequence, more effectively increases ketone body levels compared to MCTs (56). KEs, such as (R)-3-hydroxybutyl-(R)-3-hydroxybutyrate and R,S-1,3-butanediol AcAc diester, are well-tolerated, safe, and efficient ketogenic agents in both animals and humans (56, 99, 105, 106). Moreover, it was demonstrated that a proper dose of KS alone (99) or in combination with other exogenous ketone supplements, such as KE and MCT (KEKS and KSMCT, respectively), may be a safe and efficacious way to achieve ketosis (65, 75, 84, 99). Thus, exogenous ketone supplements may be an effective alternative to ketogenic diets for therapeutic ketosis.

## Therapeutic Potential of Exogenous Ketone Supplements in the Treatment of Psychiatric Diseases

Although there has been remarkable progress in our knowledge on the biological effects and mechanisms of action of exogenous ketone supplements, their exact mechanisms on CNS diseases are largely unknown. It has been demonstrated that an increase in ketone body/βHB concentration may modulate neurotransmitter balance and release (43, 52, 85), decrease hyperexcitability, reduce firing rates of neurons (43, 84, 86), decrease neuroinflammation (43, 91), enhance brain energy metabolism (43, 50, 83, 84, 87), and provide neuroprotective effects (43, 45, 84, 88, 90), which together may protect different physiological processes under pathological conditions resulting in CNS diseases, such as psychiatric disorders (35–36, 37, 58, 69). Thus, it is possible that exogenous ketone supplement-evoked ketosis (65, 75, 84) and its significant metabolic effects, as well as their consequences, may have both preventive and therapeutic potential as a metabolic-based therapy in patients with psychiatric diseases (**Figure 1B**). In spite of the several metabolic alterations, the mechanism of action of exogenous ketone supplement-evoked ketosis on different psychiatric diseases was not investigated comprehensively. As a result, we have only limited results in relation to exact links between alleviating effects of ketone supplement-generated ketosis and pathological changes in psychiatric diseases. Nevertheless, both recent literature results on basic pathomechanisms of psychiatric diseases and mechanisms of therapeutic effects of exogenous ketone supplement-evoked ketosis strongly support the hypothesis that exogenous ketone supplement-evoked ketosis may modulate the background pathophysiological processes of psychiatric diseases. Indeed, an MCT diet caused anxiolytic effects (76) and βHB decreased anxiety-related and depressive behaviors in rats and mice (114, 115). It has also been demonstrated that sub-chronic (7 days) oral administration of exogenous ketone supplements, such as KE, KS, and KSMCT, evoked an anxiolytic effect in normal rats (Sprague–Dawley/SPD rats) and diseased rats (Wistar Albino Glaxo/Rijswijk rats: WAG/Rij rats; a rat model of human absence epilepsy) on elevated plus maze (EPM) test in correlation with increased levels of βHB (75, 95). Elevated ketone body levels were demonstrated in schizophrenic patients, suggesting that the energy supply of brain shifts from glucose towards ketone bodies in this disease (116). Based on correlation between βHB plasma levels and symptoms it was suggested that βHB may have a protective effect on executive functions in patients treated with schizophrenia (117). Other studies presented cases of patients with chronic schizoaffective disorders where the KD begin helping with mood and psychotic symptoms within 1 month or lead to remission of psychotic symptoms (73, 74). It has also been suggested that plasma level of βHB is associated with severity of depression in human and that βHB-evoked antidepressant-like effects may be in relation to its inhibitory effect on NOD-like receptor pyrin domain 3 (NLRP3)-induced neuro-inflammatory processes. The authors also suggested that modification of βHB levels by diet may be a novel therapeutic target for the treatment of mood disorders, such as depression (115, 118). In addition, ketosis (induction of βHB) may be the primary mediator of the therapeutic effect of the ketogenic diet and exogenous ketone supplements on different CNS diseases. From this viewpoint, the effect of exogenous ketone supplements mimics the ketogenic diet (43, 44, 51, 52, 54, 58, 72, 94, 96, 101, 119). Thus, ketogenic diet-evoked effects on psychiatric diseases may result (at least partly) from beneficial metabolic effects of βHB, for example, on mitochondrial functions, neuronal activity, neurotransmitter release, and inflammatory processes (43, 50, 52, 86, 91). Indeed, administration of a ketogenic diet not only increased the ketone body level but also was associated with improvements in anxiety disorder (75, 77), bipolar disorder (120), schizophrenia (42, 70, 73, 74, 121), depression (77, 122), autism spectrum disorder (78, 80, 123), and ADHD (124, 125) in animal models and/or humans, suggesting the beneficial effects of exogenous ketone supplement-induced ketosis on psychiatric diseases (**Figure 1B**).

However, thorough investigation of signaling pathways by which exogenous ketone supplement-evoked ketosis exerts beneficial effects on psychiatric diseases is needed. In the following subsection, we provide an overview of the main putative basic mechanisms, by which ketone supplement-evoked ketosis may alleviate different pathophysiological processes involved in psychiatric disorders.

### Ketosis-Generated Effects on Mitochondrial Functions, Neurotransmitter Systems, Inflammatory Processes, and Their Consequences: Putative Alleviating Influences on Psychiatric Diseases

It has been demonstrated that ketone bodies serve as alternative fuel for brain cells when the glucose supply is insufficient: ketone bodies improve mitochondrial respiration and enhance mitochondrial ATP synthesis (**Figure 1B**) (47, 126). Increased mitochondrial ATP production may promote the repolarization of neuronal membrane after stimulation by means of Na^+^/K^+^ ATPase and may modulate the neurotransmitter levels (119). In addition, βHB may inhibit vesicular glutamate transporters (127). This effect, together with increased ATP production, decreases glutamate loading to vesicles and glutamate release and, as a consequence, suppresses neuronal excitability (68, 119, 127).

It was recently demonstrated that βHB inhibits the activity of N-type Ca^2+^ channels in sympathetic nerve terminals and may decrease the release of noradrenaline *via* activation of its G-protein-coupled receptor free fatty acid receptor 3 (FFAR3) (128). Increased levels of ketone bodies, such as βHB, may evoke other changes in metabolic pathways, such as inhibition of glycolysis (43). An inhibition of glycolysis may result in decreased levels of cytosolic ATP and, as a consequence, increased activity of ATP-sensitive potassium (K_ATP_) channels generating hyperpolarization of neuronal membrane and decrease in neuronal activity (43, 129). As it was demonstrated, ketosis not only decreases glutamate release and extracellular glutamate levels and enhances the GABAergic effects by means of increased GABA levels and GABA_A_ receptor activity (43, 68) but also increases adenosine levels (130) and may modulate metabolism of monoamines (**Figure 1B**). For example, increased levels of noradrenaline in mice brain (131) and decreased levels of metabolites of monoamine dopamine and serotonin (homovanillic acid/HVA and 5-hydroxyindole acetic acid/5-HIAA, respectively) in the human cerebrospinal fluid (132) were demonstrated under a ketotic state. Increased levels of extracellular adenosine lead to increased activity of adenosine receptors and may decrease hyperexcitability *via* A_1_Rs, increase hyperpolarization of neuronal membrane, and decrease neuronal activity (133, 134). In addition, adenosine decreases the energy demand of brain tissue (e.g., *via* A_1_R and A_2A_R) (135), modulates immune system functions (e.g., activation of A_2A_R decreases the inflammation-induced cytokine production from microglial cells) (136), and has a neuroprotective effect (e.g., evokes a decrease in oxidative stress and attenuates the harmful influence of ROS on brain cells *via* A_1_R) (137, 138).

β-Hydroxybutyrate may exert its effects on numerous targets, including oxidative stress mediators (e.g., by inhibition of histone deacetylases and increased activity of antioxidant enzymes) and metabolic rate (e.g., increased NAD^+^–NAD^+^/NADH ratio) directly and/or indirectly *via* its G-protein-coupled receptors, such as hydroxycarboxylic acid receptor 2 (HCAR2, also known as PUMA-G or GPR109 receptor) (45, 90, 139, 140). As an endogenous ligand, βHB activates the HCAR2 receptor expressed on, for example, microglial cells (141). HCAR2 mediates the inhibitory effects of βHB on neurodegeneration, microglial activation, and inflammatory processes [e.g., decreases the expression/level of interleukins, such as interleukin-1β (IL-1β), and lipopolysaccharide/LPS-induced increase in cyclooxygenase-2/COX-2 activity and interleukin levels] (141–143) (**Figure 1B**). NOD-like receptor pyrin domain 3 inflammasome is a multiprotein complex, which may evoke cleavage of pro-IL-1β to its active form (IL-1β) for secretion by caspase-1 (144, 145). It was demonstrated that βHB decreases inflammatory processes likely through inhibition of NLRP3: βHB decreased not only the expression of NLRP3 and caspase-1 but also the level/release of proinflammatory cytokines, such as IL-1β (91, 146).

In general, oxidative stress damages proteins, lipids, and nucleic acids. One putative downstream effect of this damage is the opening of the mitochondrial permeability transition (mPT) pore and, as a consequence, activation of the apoptotic cascade processes pursuant to release of cytochrome c to the cytoplasm (147). It was demonstrated that increased production of ROS may activate mPT pore (97, 147). Ketone bodies decreased oxidative stress and ROS formation by enhancing complex I (NADH dehydrogenase)-driven mitochondrial respiration (140). It has also been demonstrated that KE increased both ketone body levels and expression of mitochondrial uncoupling proteins (UCPs; e.g., UCP 4 and UCP 5 in rat brain), which can decrease the production of ROS (50, 148) (**Figure 1B**). In addition, it was suggested that βHB not only prevents neuronal loss but also preserves synaptic function: βHB mitigates effects, which may evoke cell death/apoptosis (e.g., glutamate excitotoxicity, enhanced ROS production, impaired mitochondrial energetic functions, pathogenic mutations on mitochondrial DNA, and activation of mPT pore) (44, 97, 119, 149), and βHB may restore impairment of hippocampal long-term potentiation (150).

The changes induced by ketosis may lead to enhanced brain energy metabolism, promotion of repolarization of neuronal membrane, neuronal hyperpolarization, decreased hyperexcitability and neuronal firing, modulation of neurotransmitter release/balance, neuroprotective effects, and decreased inflammatory processes (**Figure 1B**). Downstream effects may include increased GABA and ATP/adenosine levels, decreased levels of glutamate and IL-1β, and reductions in neuronal excitability and ROS formation. Based on these putative alleviating effects, which may have therapeutic potential in the treatment of different psychiatric diseases, this subsection is followed by a brief overview of the main pathological changes in different psychiatric diseases, which may be modulated or improved by ketosis-evoked beneficial effects and their consequences. Currently, we lack detailed information for understanding the exact mechanisms by which ketosis evokes beneficial effects on psychiatric disorders. However, we can be reasonably confident that the alleviating effects of exogenous ketone supplements on these disorders affect several interacting factors, including mitochondrial function, neurotransmitter levels, and inflammatory processes.

#### Anxiety Disorders

An increasing body of evidence suggests that dysregulation of the glutamatergic, serotonergic, purinergic, and GABAergic systems plays a role in the pathophysiology of anxiety disorders (33, 34, 151–153). For example, inhibition of NMDA and AMPA receptors by their antagonists (e.g., DL-2-amino-5-phosphonovaleric acid/APV and 6-cyano-7-nitroquinoxaline-2,3-dione/CNQX, respectively) fully or partially blocked the expression and/or acquisition of fear conditioning (30, 154). Activation of the serotonergic system (e.g., *via* increased levels of serotonin by selective serotonin reuptake inhibitors/SSRIs and activation of serotonin 5-HT1A receptors by buspirone or tandospirone) and increased activity of adenosinergic system (e.g., *via* activation of A_1_ type of adenosine receptors/A_1_R) have an anxiolytic effect (34, 155). Moreover, enhanced GABAergic neurotransmission evoked an anxiolytic effect, whereas decreased GABAergic transmission generated anxiogenic responses in animals (151, 153, 156). Altered functions are present in many regions, including the extended amygdala, ventromedial prefrontal cortex, hippocampus, hypothalamus, and the midbrain, and changed connections between these areas are implicated in the pathophysiology of anxiety disorders (157–159). Specific changes, such as underactivation (e.g., in ventromedial prefrontal cortex), overactivaton (e.g., in amygdala), and deficient functional connectivity (e.g., between hippocampus and amygdala), have also been demonstrated (157, 158, 160, 161). Changes in gray matter volume (e.g., in the right orbitofrontal cortex, amygdala, and hippocampus) (160, 162, 163), as well as dysfunction or hyperactivation of HPA axis and inflammatory system (e.g., increased level of proinflammatory cytokines) (14, 164), may have a role in pathophysiology of anxiety disorders. It was also demonstrated that mitochondrial dysfunctions and oxidative stress may be key factors in the emergence of anxiety disorders (165, 166).

#### Schizophrenia

It has been demonstrated that alterations in the neurotransmitter systems governed by GABA, glutamate, and the monoamines are involved in the development of schizophrenia (7, 23, 27, 32, 167–169). For example, in the prefrontal cortex, which partially mediates the negative symptoms of schizophrenia, low serotonin and dopamine levels were detected (7, 23). Cognitive symptoms may be linked with decreased level of GABA and serotonin (e.g., in the dorsolateral prefrontal cortex) (7, 170). Moreover, decrease in serotonin level was demonstrated in amygdala, which may lead to aggressive symptoms (7). It was concluded that, among others, hypofunction of the inhibitory GABAergic interneurons and changes in activity of implicated brain areas (e.g., because of decreased activity of inhibitory effects and imbalance between inhibitory/excitatory processes) have a role in the pathophysiology of schizophrenia (7, 167). Another recent study using an acute NMDA receptor hypofunction model of schizophrenia showed that feeding C57BL/6 mice a low carbohydrate/high-fat KD for 7 weeks prevented a variety of behavioral abnormalities induced by pharmacological inhibition of NMDA glutamate receptors (42). In the study, they found a lack of correlation between the measured prepulse inhibition of startle and body weight changes, providing evidence against the role of calorie restriction in its mechanism of action (42). Case studies on human patients with schizophrenia also supported the efficacy of using KD to improve symptoms (73, 74). Reduction in the volume of brain areas encompassing cortical gray and white matter (e.g., in amygdala and hippocampus/sensorimotor and dorsolateral prefrontal cortices) (171–173), gliosis (174), and increased neuronal apoptosis (7, 175) were also demonstrated in patients with schizophrenia. A great deal of evidence suggests that microglial activation, oxidative stress (e.g., increase in ROS activity), and mitochondrial dysfunction (e.g., changes in activity of complex I and cytochrome-c-oxidase/IV of electron transport chain) may also be involved in the pathophysiology of schizophrenia (167, 176–178). Increased activation of HPA axis by psychological stress, inflammatory processes, and increased level of cytokines (e.g., tumor necrosis factor alpha/TNF-α and IL-1β), as well as enhanced levels of glutamate and dopamine auto-oxidation, could lead to enhanced production of ROS and subsequently neurodegeneration and apoptosis (7, 167, 178–180).

#### Major Depressive Disorder

Structural brain alterations, such as decreased volume and cell number of brain areas (e.g., in hippocampus and several cortical areas) (3, 181–183) and abnormalities in activation or connectivity of brain structures and networks (e.g., chronic hyperactivity of limbic centers and brainstem) (13, 22, 184–186), may underlie the functional and behavioral changes observed in depressed patients. It has been demonstrated that changes in several components, including the glutamatergic system (e.g., increased glutamate level) (29), monoaminergic system (e.g., decrease in the level of serotonin, noradrenaline, and dopamine) (3, 13, 24, 187, 188), GABAergic system (e.g., reduced plasma and cerebrospinal fluid GABA levels) (189, 190), and purinergic system (e.g., overexpression of A_2A_ type of adenosine receptors/A_2A_R) (28) have a role in the pathophysiology of major depressive disorder. Activation of microglia and astrocytes and inflammatory pathways (14, 164, 191, 192) may be associated with major depressive disorder. For example, increased activation and expression of NLRP3 inflammasome and interleukins (e.g., IL-1β) were revealed in both animal models and patients with depression (13, 193, 194). Hyperactivity of HPA system was also demonstrated (195). Neurodegeneration and neuronal death (e.g., through increased oxidative/nitrosative stress) and alterations in mitochondrial functions (e.g., decreased ATP production as well as enhanced apoptosis and oxidative stress) (35, 177, 196) also play a role in the emergence of major depressive disorder. It has been demonstrated that enhancement of inflammatory processes is associated with depression by modulation of different neurotransmitter systems: for example, inflammatory cytokines (e.g., IL-1β) reduce synaptic availability of monoamines and increase excitotoxicity (*via* extrasynaptic NMDA receptors) by increasing levels of extracellular glutamate (164, 197, 198). Moreover, cytokines may evoke decreased motivation and anhedonia *via* different pathways (e.g., by decreased release of dopamine in the basal ganglia) (164, 199).

#### Bipolar Disorder

It has been demonstrated that imbalance in monoaminergic neurotransmitter system (e.g., serotonergic, dopaminergic, and noradrenergic) (200–202), GABAergic system (e.g., decreased GABAergic transmission) (190), purinergic system (e.g., increased level of uric acid and reduced adenosinergic activity at A_1_Rs) (31), and glutamatergic system (e.g., increased glutamate levels and NMDA receptor activity) (29) are associated with bipolar disorder. These alterations may be associated with mitochondrial dysfunction (e.g., deficit in activity of complexes I and IV), apoptosis, increase in ROS, oxidative damage, hyperexcitability (5, 177, 203, 204), and, as a consequence, decrease in glial cell or neuron number and gray matter, as well as changes in connectivity between implicated brain areas (e.g., hippocampus, prefrontal cortex, and amygdala) (205–207). Changes in endocrine functions (e.g., dysregulation of HPA axis) and inflammatory processes (e.g., increased proinflammatory cytokine levels, such as IL-1β) were demonstrated in association with bipolar disorder (203, 208).

#### Autism Spectrum Disorder

It has been demonstrated that agenesis of corpus callosum, changes in brain volume, thinning of several brain cortical areas (e.g., in the frontal parietal lobe), and decreased functional connectivity between brain areas (e.g., within frontal cortex) contribute to pathophysiology of autism spectrum disorder (209–212). It was also demonstrated that dysfunction in glutamatergic system (e.g., exaggerated signaling) (213–215) and GABAergic system (e.g., decreased GABA receptor expression and GABA-evoked inhibitory effects) (215, 216) may have a role in the pathophysiology of autism spectrum disorder by alterations in the excitation/inhibition balance. In addition, decreased level of serotonin/adenosine in implicated brain areas (e.g., medial frontal cortex) have also been demonstrated/suggested in this disease (25, 217–220). Impaired immune response, inflammation, and oxidative stress may be causative factors of autism spectrum disorder (15, 221). In fact, recent studies suggest that autism spectrum disorder is associated with inflammation (e.g., activation of glial cells and increased levels of cytokines) (222–224), mitochondrial dysfunction, and oxidative stress (e.g., increased ROS activity) (79, 225–227).

#### Attention Deficit/Hyperactivity Disorder

Reduction of brain volume and gray matter (e.g., in putamen and caudate nucleus) and underactivation or hyperactivation of different brain networks (e.g., in the frontoparietal and ventral attention network and the somatomotor system) were demonstrated in patients with ADHD (228, 229). Numerous studies have shown that increased glutamatergic tone/glutamate level (230), dopamine hypofunction (e.g., decreased stimulation-evoked release of dopamine) (26), and changes in GABAergic (e.g., decrease in GABA level) (230, 231), noradrenergic, and serotonergic system (16, 232–235) in the implicated brain areas may be causative factors of ADHD. Furthermore, increased oxidative stress (e.g., enhanced production of ROS) was demonstrated in a rat model of ADHD (236).

## Conclusion

The effects of nutritional ketosis on CNS diseases, whether through diet or supplementation, have not been fully investigated. Consequently, only limited results have demonstrated the existence of alleviating effects of exogenous ketone supplement administration on animal models of psychiatric diseases and patients with psychiatric disorders. Nevertheless, there are several common pathophysiological metabolic alterations, such as changes in neurotransmitter release, increased inflammatory processes, abnormal cerebral glucose metabolism, and decreased mitochondrial-associated brain energy metabolism, which may have a role in the emergence of psychiatric diseases. Consequently, ketogenic interventions that can modulate a broad array of metabolic and signaling changes underlying the pathophysiology of psychiatric diseases may alleviate the onset of symptoms.

Based on our review of the literature, we hypothesize that utilizing exogenous ketone supplements alone or with ketogenic diet, either as a primary or an adjunctive therapy for selected psychiatric disorders, may potentially be an effective treatment. Thus, adding ketone supplements as an additional agent to the therapeutic regimen may alleviate symptoms of psychiatric diseases *via* modulation of different metabolic routes implicated in psychiatric disorders. Therefore, detailed investigation of exogenous ketone supplement-evoked direct and/or indirect alterations in molecular pathways and signaling processes associated with psychiatric diseases is needed.

The use of exogenous ketone supplements in psychiatric diseases is only in its infancy. Nevertheless, our increasing understanding of how exogenous ketone supplement-evoked ketosis/βHB exerts its effects on CNS diseases, combining with new results on pathophysiology of psychiatric diseases and their complex interplay with each other, suggests that exogenous ketone supplements may be ideal and effective adjuvants to drugs used in the treatment of psychiatric diseases. Thus, because exogenous ketone supplements modulate endogenous processes, their administration is a safe method to promote disease-alleviating effects without considerable risk, as well as minimal or no side effects compared to pharmacological treatments. Consequently, exogenous ketone supplements may help to both manage the side effects and increase the efficacy of drugs used in psychiatric diseases, especially in cases of treatment resistance.

Future research should explore the effects of exogenous ketones on the metabolic processes that underlie the diseases leading to psychiatric disorders in order to restore abnormal cerebral glucose and energy metabolism. Moreover, new studies are needed to investigate the effects, therapeutic efficacy, and exact mechanism(s) of action of exogenous ketone supplements alone or in combination with a ketogenic diet not only on animal models of psychiatric diseases, but also on patients with different psychiatric disorders. Future studies are needed to reveal which factors (e.g., age, sex, lifestyle, drugs, other diseases, and so on) can modify the effects of exogenous ketone supplements on psychiatric diseases; to develop new, more effective, and safe ketone supplements, which can be used in special ketogenic foods for treatment of CNS disorders, including psychiatric diseases. There is urgent need to develop therapeutic strategies and broadly accepted protocols guiding the administration of different types and combinations of exogenous ketone supplements. As a result of new studies in the near future, a better understanding of the pathophysiology of different psychiatric diseases and the connections between the underlying metabolic/signaling pathways may promote the development of novel metabolism-based adjuvant therapies, such as the administration of exogenous ketone supplements against psychiatric diseases.

## Author Contributions

ZK contributed to the conception of the manuscript, comprehensive search of the electronic databases, and writing of the manuscript. DPD contributed to the writing of the manuscript. DD, MK, and CR were in charge of revising the manuscript. CA was in charge of writing and revising the manuscript.

## Conflict of Interest Statement

International Patent #PCT/US2014/031237, University of South Florida, D.P. D’Agostino, S. Kesl, P. Arnold, “Compositions and Methods for Producing Elevated and Sustained Ketosis.” Non-provisional patents: #62289749, University of South Florida, C. Ari, D.P. D’Agostino, “Exogenous ketone supplements for reducing anxiety-related behavior”; Ari, C., Arnold P., D’Agostino, D.P. Technology Title: “Elevated blood ketone levels by ketogenic diet or exogenous ketone supplements induced increased latency of anesthetic induction” USF Ref. No. 16A018PR; Ari, C., Arnold P., D’Agostino, D.P. Technology Title: “Exogenous ketone supplementation improved motor function in Sprague–Dawley rats.” USF Ref. No: 16A019; Ari, C., Arnold P., D’Agostino, D.P. Technology Title: “Lowering of blood glucose in exercising and non-exercising rats following administration of exogenous ketones and ketone formulas.” USF Ref. No: 16A049; Ari, C., Arnold P., D’Agostino, D.P. Technology Title: “Ketone supplementation elevates blood ketone level and improves motor function in GLUT1 deficiency syndrome mice.” USF Ref. No: 16B116 (provisional patent); Ari, C., Arnold P., D’Agostino, D.P. Technology Title: “Neuroregeneration improved by ketone.” USF Ref. No: 16B128 (provisional patent); Ari, C., D’Agostino, D.P. Dean, J.B. Technology Title: “Delaying latency to seizure by combinations of ketone supplements.” USF Ref. No: 16B138PR. D.P. D’Agostino and C. Ari are co-owners of the company Ketone Technologies LLC, providing scientific consulting and public speaking engagements about ketogenic therapies. The company obtained an option agreement from the University of South Florida on the non-provisional patent no. 62/310,302 “Methods of increasing latency of anesthetic induction using ketone supplementation.” These interests have been reviewed and managed by the University in accordance with its Institutional and Individual Conflict of Interest policies. All authors declare that there are no additional conflicts of interest.

## Abbreviations

A_1_R and A_2A_R, different types of adenosine receptors; AcAc, acetoacetate; ADHD, attention-deficit/hyperactivity disorder; BBB, blood–brain barrier; βHB, D-beta-hydroxybutyrate (R-3-hydroxybutyrate); β-OHBD, βHB dehydrogenase; CNS, central nervous system; COX-2, cyclooxygenase-2; FFAR3, free fatty acid receptor 3; HCAR2, hydroxycarboxylic acid receptor 2; HPA, hypothalamic-pituitary-adrenal; HMG-CoA, hydroxymethylglutaryl-CoA; HMGL, hydroxymethylglutaryl-CoA-lyase; HMGS, hydroxymethylglutaryl-CoA-synthase; KE, ketone ester; KS, ketone salt; IL-1β, interleukin-1β; MCT, medium chain triglyceride; NLRP3, NOD-like receptor pyrin domain 3; ROS, reactive oxygen species; SCOT: succinyl-CoA:3-ketoacid CoA transferase; SSRI, selective serotonin reuptake inhibitor; UCP, uncoupling proteins; WAG/Rij, Wistar Albino Glaxo/Rijswijk.
